# Coastal upwelling generates cryptic temperature refugia

**DOI:** 10.1038/s41598-022-23717-5

**Published:** 2022-11-11

**Authors:** Sarah L. Salois, Tarik C. Gouhier, Brian Helmuth, Francis Choi, Rui Seabra, Fernando P. Lima

**Affiliations:** 1 Marine Science Center, Northeastern University, 430 Nahant Road, Nahant, MA 01908 USA; 2 CIBIO, Centro de Investigação em Biodiversidade e Recursos Genéticos, InBIO Laboratório Associado, Universidade do Porto, Campus de Vairão, 4485-661 Vairão, Portugal; 3 BIOPOLIS Program in Genomics, Biodiversity and Land Planning, Campus de Vairão, 4485-661 Vairão, Portugal; 4 Present Address: School for Marine Science and Technology, University of Massachusetts Dartmouth, 836 South Rodney French Blvd, New Bedford, MA 02744 USA

**Keywords:** Ecology, Climate-change ecology, Physical oceanography

## Abstract

Understanding the effects of climate-mediated environmental variation on the distribution of organisms is critically important in an era of global change. We used wavelet analysis to quantify the spatiotemporal (co)variation in daily water temperature for predicting the distribution of cryptic refugia across 16 intertidal sites that were characterized as ‘no’, ‘weak’ or ‘strong’ upwelling and spanned 2000 km of the European Atlantic Coast. Sites experiencing weak upwelling exhibited high synchrony in temperature but low levels of co-variability at monthly to weekly timescales, whereas the opposite was true for sites experiencing strong upwelling. This suggests upwelling generates temporal thermal refugia that can promote organismal performance by both supplying colder water that mitigates thermal stress during hot Summer months and ensuring high levels of fine-scale variation in temperature that reduce the duration of thermal extremes. Additionally, pairwise correlograms based on the Pearson-product moment correlation coefficient and wavelet coherence revealed scale dependent trends in temperature fluctuations across space, with a rapid decay in strong upwelling sites at monthly and weekly timescales. This suggests upwelling also generates spatial thermal refugia that can ‘rescue’ populations from unfavorable conditions at local and regional scales. Overall, this study highlights the importance of identifying cryptic spatiotemporal refugia that emerge from fine-scale environmental variation to map potential patterns of organismal performance in a rapidly changing world.

## Introduction

Predicting species distributions and ascribing them to their underlying causes is a key component of conservation and climate adaptation strategies^1^. However, identifying the mechanisms behind patterns of species abundance is particularly challenging, as natural systems are coupled in space and time via the interplay of eco-environmental feedbacks operating at multiple scales. This inherent complexity has led to fundamental debates about the scales and approaches that are most likely to yield fundamental insights about ecological systems^2–4^.

For instance, proponents of macroecology suggest that the idiosyncratic and system-specific results that often plague community studies conducted at small scales give way to statistically consistent patterns at larger scales^2^. While macroecological studies are useful for identifying patterns that hold across large swaths of taxa and systems over large temporal and spatial scales, they are often unable to link these general relationships to their causal mechanisms. For instance, a review by McGill et al.^5^ highlighted dozens of mechanisms ranging from diametrically opposed niche and neutral assembly processes that could give rise to the “hollow curve”, the quasi-universal distribution of abundance whereby communities are dominated by a few abundant species and a multitude of rare ones. Hence, the search for universality can be characterized as a double-edged sword because predictability often comes at the cost of mechanistic understanding.

Conversely, community ecology, which has traditionally focused on small scales, has had a successful track record of using manipulative experiments to identify the mechanistic underpinnings of many ecological patterns^3^. However, ‘scaling-up’ these results to understand species-rich ecosystems across biogeographic scales is rarely feasible, so a majority of the work has focused on attempting to reduce the dynamics of these inherently complex systems to those of simple community modules consisting of a few key species (e.g., keystone species^6^, or foundational species^7^) and interactions (e.g., trophic cascades^8^). Unfortunately, the diversity of species and interactions that characterize natural systems makes it hard to ‘scale-up’ from simple communities to whole ecosystems^9,10^. Hence, while community ecology can often reveal the mechanisms that underpin local patterns at any one particular site, these relationships often fail to hold across ecosystems and scales^4^.

More recently, ecologists have developed multi-scale approaches that bridge the gap between macroecology and community ecology in order to understand how feedbacks between local and regional processes give rise to ecological communities^11–13^. For instance, metacommunity ecology has demonstrated that even in the presence of strong regional processes, species interactions can have significant impacts on the demographic properties of local populations and, in doing so, generate dynamical signals that propagate across scales via dispersal to control the spatial structure of entire metacommunities^14–16^. These multi-scale frameworks demonstrate that fine-scale ecological and environmental processes are critical for predicting the dynamics of populations in space and time.

Integrating the effects of fine-scale environmental variation is particularly important in order to produce a better understanding of organismal performance under climate change^17–19^. In turn, this knowledge can be used to inform management and assessment of important ecosystems^20^. Modeling approaches that assume stationary and linear relationships between populations and their environment are likely to fail under the novel suites of environmental conditions created by climate change^21^. For instance, bioclimate envelope models often generate predictions about how the distribution of species will shift under future climatic conditions by using contemporary relationships between population abundances and environmental variables at coarse spatial and temporal scales^22,23^. Despite their power and simplicity, these approaches make assumptions that may fundamentally limit their forecasting ability in most systems^18,24,25^. Specifically, measuring environmental variables at coarse spatial and temporal scales can mask important variation that could influence population and community dynamics^26–29^. Helmuth et al.^26^ showed this empirically when they revealed the complex and counterintuitive way fine-scale measurements of intertidal organismal body temperature differed greatly from those predicted by broad-scale climatic patterns. Kroeker et al.^30^ further demonstrated the influence of mosaic patterns of multiple environmental drivers (ocean pH, temperature and chlorophyll) in determining biogeographic patterns of growth and reproduction. Additionally, assuming that fine-scale spatial variation in climatic and environmental variables does not affect ecological patterns at greater scales ignores evidence that cross-scale interactions between regional dispersal and local processes can give rise to large-scale patterns^14,15^.

The temporal scale and structure of climatic and environmental variables can also play a critical role when attempting to forecast population dynamics. Indeed, disregarding the temporal characteristics of key climatic factors, such as temperature, can oversimplify environmental stressors by reducing them to averages, thus ignoring meaningful variability that can dictate organismal performance and survivorship^29,31,32^. Additionally, averaging-out fine-scale spatial variation in environmental conditions can exacerbate these issues by also altering their temporal structure and thus masking spatiotemporal refugia, broadly defined as areas which promote the persistence of organisms under changing environmental conditions^33–35^. The identification of climate refugia has been based primarily on measurements of climate velocity, i.e., long-term changes in mean conditions^36^. This approach is an important first approximation and could be extended by considering other statistical moments in climate conditions. In particular, spatial and temporal refugia associated with environmental variation can promote persistence at local and regional scales via variation in recruitment, buffered population growth and other mechanisms that promote spatial rescue effects^37–39^, all of which may not be captured by temporally-averaged data^40^. Recent evidence highlights the importance of characterizing environmental variation at the scales and resolutions relevant to the organisms of interest when attempting to predict the effects of environmental change^41,42^.

An important consequence is that the scales over which environmental data are collected can unintentionally bias our understanding of the actual scales of meaningful variability in nature^35,43^. For example, while data from remote sensing platforms remain the best way to obtain global coverage, they cannot capture the often very high within-pixel variability in factors such as temperature^44,45^. This heterogeneity is particularly true in coastal zones. Multiple studies have shown that onshore water temperatures can be very different from nearshore temperatures recorded by buoy or satellite^43,46,47^, and others have shown consistent differences between sea surface temperature (SST) and temperature at depth^48,49^. While some of these differences are due to heating of shallow waters by solar radiation, at other sites this appears to be driven by formation of internal waves^50^ or near-shore upwelling, a process that brings cold and nutrient-rich waters to the surface of the coastal ocean^46,47^. The end result is that while regions of upwelling can potentially serve as refugia by reducing thermal stress^51–53^, their cold signature is not always detected via measurements of nearshore SST from satellites^47,54,55^.

Here, we addressed the importance of scale for understanding the potential for species responses to environmental conditions via the identification of thermal refugia. Specifically, we examined the effects of daily temperature variation, obtained from autonomous sensors deployed at a series of intertidal sites, on the emergence of cryptic temporal and spatial refugia associated with an upwelling system. This dataset provided more than seven years of daily onshore water temperatures along approximately 20 degrees of the European Atlantic Coast that ranged from the Atlantic Iberian coast, which is part of the northern section of the Iberia/Northwest Africa Eastern boundary upwelling ecosystem known as the Canary Current System, to the British Isles. This served as a model system to investigate the impacts of environmental variability on the potential for species persistence, as intertidal ecosystems are characterized by severe environmental forcing via strong temperature fluctuations and extremes. These fluctuations are a product of tidal influences (resulting in zonation) and geographic location (juxtaposition between the land and sea), and have resulted in strong and well documented relationships between thermal stress, fitness and survivorship for the organisms that live there^19,56,57^. Additionally, the oceanographic variability associated with upwelling systems^58,59^ makes them model regions to examine how wind-driven currents can alter the temperatures experienced at organismal scales now and in the future, as their duration and intensity are expected to increase under climate change^52,60^.

**Figure 1:**
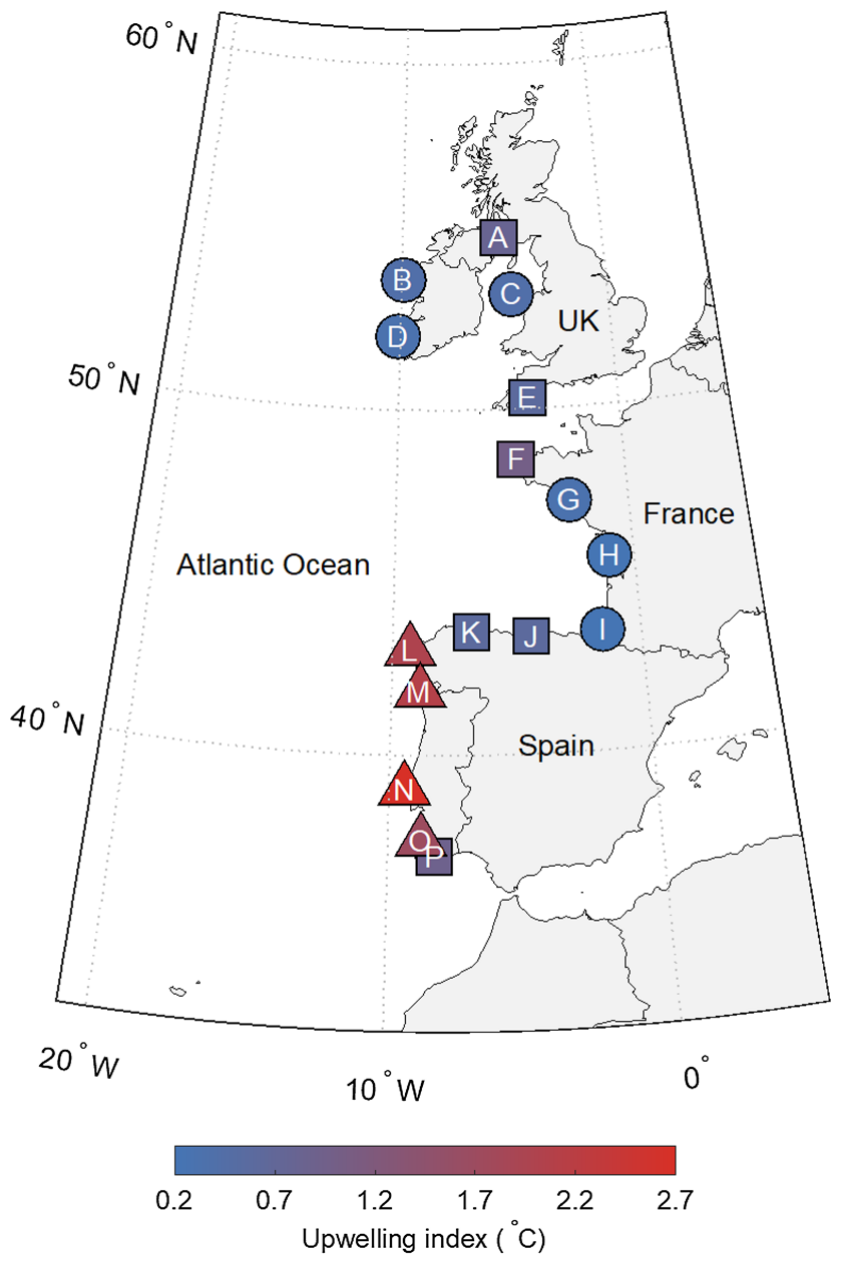
Map of the study sites. Letters A-P indicate the locations of the 16 sites where temperature logger data were collected, which covers the broad range of *Patella vulgata’s* southern distribution. Sites are labeled in descending order from Northernmost to Southernmost latitudes, where: A—South Cairn, UK; B—Emlagh, IE; C—Anglesey, UK; D—Minard Castle, IE; E—Wembury, UK; F—Landunvez, FR; G—Le Croisic, FR; H—Royan, FR; I—Biarritz, FR; J—San Vicente de la Barquera, ES; K—La Caridad, ES; L—Cabo Touriñan, ES; M—Moledo, PT; N—São Lourenço, PT; O—Alteirinhos, PT; P—Evaristo, PT. Colors represent the thermal upwelling index, which ranges from low upwelling (blue) to strong upwelling (red). Circles represent sites characterized by no upwelling (0.0–0.4), squares represent weak upwelling (0.5–1.0), and triangles represent strong upwelling (1.1–3.0). Map was created in MATLAB, version 9.11^108^.

The goal of our study was to evaluate intertidal (onshore) water temperature at different spatiotemporal scales to determine how scale can influence the interpretation of environmental trends and the subsequent identification of thermal refugia in space and time. Specifically, we used pairwise correlations and wavelet coherence of onshore water temperature across 16 sites along the European Atlantic Coast (Fig. 1) to examine how temperature varied within and between regions characterized by differential upwelling condition. We found that using simple raw time series alone masked the underlying relationships between water temperature and upwelling strength, whereas upwelling-dependent spatiotemporal refugia emerged when the same data were analyzed at ecologically relevant timescales. These results highlight the importance of temporal scale when predicting the organismal effects of climate-mediated changes in the environment.

## Materials and methods

### Onshore water temperature dataset

Analyses were conducted on a long-term dataset of daily intertidal water temperatures recorded via biomimetic temperature loggers (robolimpets; see Lima and Wethey^61^) along the European Atlantic Coast (Fig. 1). Robolimpets are autonomous temperature sensing devices designed to mimic the thermal characteristics of the common intertidal limpet, *Patella vulgata*. To that end, the device comprises a lithium battery connected to a circuit board and encased inside an empty limpet shell (see Lima and Wethey^61^ for a more detailed description). Like other biomimetic temperature loggers^62^, robolimpets are specifically designed to record temperatures comparable to those experienced by animals during aerial exposure at low tide. However, by parsing data into periods of aerial exposure and submersion, they have the additional benefit of providing onshore water temperature measurements at ecologically relevant scales^63^. Specifically, we analyzed the average daily water temperature at the peak of high tide for loggers located in the mid zone of 16 wave-exposed shores, spanning latitudes ranging from 37° to 55° N (as described by Seabra et al.^64^ and Lima et al.^65^, see Fig. 1, Table 1). We truncated the resulting time series from each location to a common seven-year time period from July 2010 to August 2017.

**Table 1:** Coastal locations of (in situ) water temperature measurements used in this study. The sites were selected to represent the broad range of *Patella vulgata’s* southern distribution. Temperatures were recorded by Maxim DS1922 iButton loggers deployed in the intertidal at 0–1 m below Mean Sea Level (MSL) at a particular frequency (in minutes, t_x_). Daily values were obtained by averaging data collected by one of multiple sensors (N loggers). These in situ measurements were used to calculate a Thermal Upwelling Index (UI, in °C) for each location, defined as the magnitude of the cold anomaly (i.e. cold bias) between upwelled coastal waters and the warmer offshore waters^47,66^. Manufacturer reported logger accuracy and resolution for the iButton loggers were both 0.50 °C.

Country	Location	Lat (degrees)	Lon (degrees)	t_x_ (min)	N loggers	UI (°C)	Classification
Scotland	South Cairn	54.97	− 5.18	60	18	0.8	Weak upwelling
Ireland	Emlagh	53.75	− 9.91	60	18	0.4	No upwelling
Wales	Isle of Anglesey	53.32	− 4.66	60	18	0.4	No upwelling
Ireland	Minard Castle	52.13	− 10.11	60	18	0.3	No upwelling
England	Wembury	50.31	− 4.11	60	18	0.6	Weak upwelling
France	Landunvez	48.54	− 4.75	60	18	1.0	Weak upwelling
France	Le Croisic	47.29	− 2.54	60	18	0.3	No upwelling
France	Royan	45.61	− 1.03	60	18	0.2	No upwelling
France	Biarritz	43.48	− 1.56	60	18	0.2	No upwelling
Spain	San Vicente de la Barquera	43.41	− 4.44	60	18	0.6	Weak upwelling
Spain	La Caridad	43.57	− 6.83	60	18	0.6	Weak upwelling
Spain	Cabo Tourinan	43.04	− 9.29	60	18	2.0	Strong upwelling
Portugal	Modelo	41.84	− 8.87	60	18	2.1	Strong upwelling
Portugal	São Lourenço	39.01	− 9.42	60	18	2.7	Strong upwelling
Portugal	Alteirinhos	37.52	− 8.79	60	18	1.7	Strong upwelling
Portugal	Evaristo	37.07	− 8.30	60	18	0.9	Weak upwelling

The locations of the sites used in this study span nearly 20 degrees of latitude from Southern Portugal to Southwest Scotland (Fig. 1, A:P). In order to capture the upwelling gradient along the European Atlantic coast, five sites were located within and eleven sites were located outside the Canary Current System, one of the four coastal upwelling regions classified as an Eastern Boundary Upwelling System (EBUS). Coastal upwelling is defined by a well-described oceanographic process where winds displace surface waters, creating an upward movement of nutrient-rich cold water. The process of ‘upwelling’ nutrients to the surface creates highly productive ecosystems, distinguishing these regions as critically important both ecologically and economically. These upwelling events prevail from May to September in this EBUS whereas during the rest of the year, the inverse process (downwelling) dominates the system. The myriad of mesoscale and submesoscale physical processes at play in coastal systems lead to high spatial heterogeneity both within and across upwelling systems. Onshore water temperature represents the outcome of the complex interaction between these complex and variable features. Therefore, while upwelling is not the sole cause of the variability in these coastal regions, it is a major contributor and examining onshore water temperatures across a series of coastal sites that span a range of upwelling intensities provides insights on the significance of temperature variability for resident intertidal organisms. Specifically, sites used in this study were assigned a Thermal Upwelling Index (UI) value ranging from 0.2 to 2.7. The upwelling condition at each site was then labeled as ‘no’, ‘weak’, or ‘strong’ based on the UI value (no upwelling: 0.0–0.4, weak upwelling: 0.5–1.0, strong upwelling: 1.1–3.0, see Fig. 1 and Table 1). This classification system is consistent with site descriptions found in the literature^64^. The UI was computed following the methods of Meneghesso et al.^47^, which used a slightly modified version of the temperature-based ‘integrated anomaly’ index described by Tapia et al.^66^. The UI considers both the duration and the intensity of thermal anomalies associated with the uplift of cold water and is defined as the cold anomaly between upwelled coastal waters and the warmer offshore waters. Coastal water temperatures were obtained from in situ loggers, and the corresponding offshore temperatures were derived from monthly gridded summaries of the International Comprehensive Ocean–Atmosphere Data Set (ICOADS, https://icoads.noaa.gov)^67^, at one arc-degree resolution. Given the coarse spatial resolution of ICOADS data, linear latitudinal transects ~ 200 km offshore from each coastline were used to compute linear regressions between monthly ICOADS temperatures and latitude. The linear regression estimators were then used to obtain monthly offshore SST outside the bulk of the influence of coastal upwelling at each study site and date. UI was computed as the mean absolute difference between the daily in situ onshore water temperatures and the corresponding latitude-corrected monthly offshore SST whenever coastal waters were cooler. A value of 2 °C, for instance, indicates that upwelling events, on average, reduced coastal water temperature by 2 °C relative to offshore waters. It is important to note that the results presented in the main text were robust to an alternative classification scheme wherein three sites were relabeled as ‘no upwelling’ based on independent oceanographic information (sites ‘A’ [South Cairn, Scotland], ‘E’ [E. Wembury, England], and ‘F’ [Landunvez, France]; see Appendix S4).

### Detecting spatiotemporal trends in onshore water temperature

**Figure 2:**
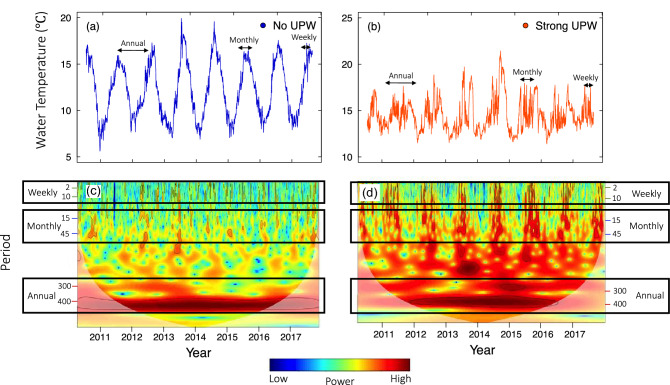
Time series and wavelet analysis of onshore water temperature across a site characterized by no upwelling (**a**, **c**) and strong upwelling (**b**, **d**). Wavelet power quantifies the relative contribution of variation in water temperature at each period to the total variance over time. This study compares annual and subannual periodicities to investigate a range of temporal scales that are more relevant to marine organisms (weekly, monthly). High power is represented in warm colors and low power is represented in cool colors. Black contours designate regions of significantly high temporal variation compared to a null model (red noise). The bolder color cone shape represents the cone of influence—values outside of this region are less reliable due to edge effects.

In order to fully document the spatiotemporal trends in onshore water temperature, we analyzed the daily water temperature time series using classical methods resolved in time only as well as wavelet methods capable of analyzing the time series in both the time and frequency domains (Fig. 2). This approach allowed us to gain insights into the processes that gave rise to the spatiotemporal patterns of temperature variation across the Eastern Atlantic Coast.

### Identifying the drivers of temporal patterns

To quantify the potential for temporal thermal refugia, defined here as areas of thermal refuge in time, we calculated the degree of synchrony and co-variability of onshore water temperatures between sites both within and across upwelling conditions over time.

#### Raw time series analysis

**Figure 3:**
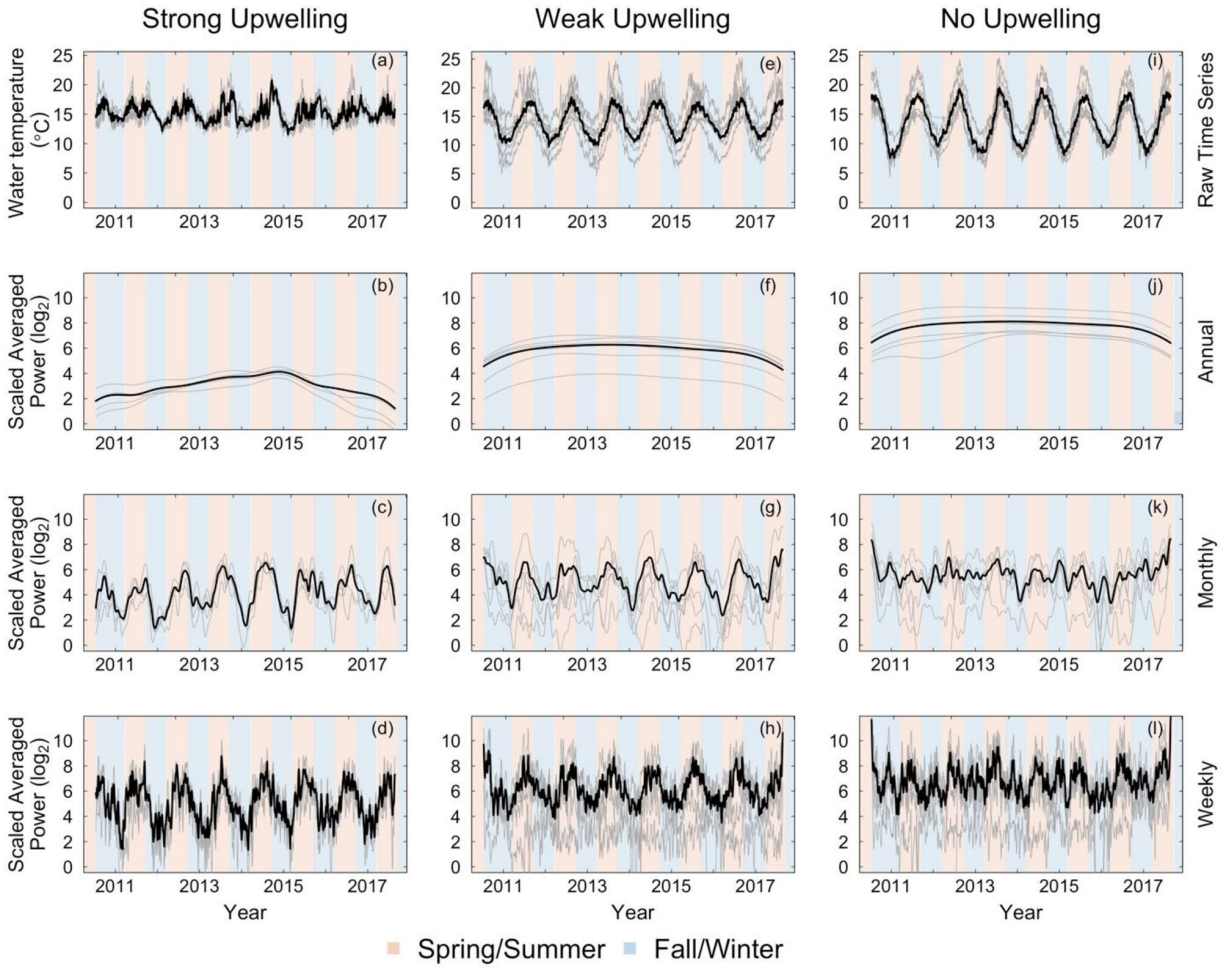
Time series and scale-averaged wavelet power for daily onshore water temperature at all sites. Columns represent site groupings based on upwelling strength (i.e., strong upwelling: 1.1–3.0, weak upwelling: 0.5–1.0, no upwelling: 0.0–0.4). The first row depicts the raw time series, and subsequent rows represent scale-averaged power at annual (300–400 days), monthly (15–45 days) and weekly (2–10 days) periodicities, respectively. Time series for individual sites are represented by grey lines and bold black lines depict the mean time series averaged across all sites. The background color indicates Spring/Summer months (orange) or Fall/Winter months (blue).

All time series were truncated to a common time frame (07-18-2010 to 08-31-2017) and grouped based on the thermal upwelling index (ranging from 0.2 to 2.7) associated with each site^64,68,69^. Daily water temperature values were plotted as a function of time for each individual site as well as averaged across sites for each upwelling condition (Fig. 3, row 1). Kendall’s Coefficient of Concordance (*W*) was calculated to measure the degree of synchrony and co-variability between sites within each upwelling condition. Kendall's *W* is a non-parametric statistic that ranges from 0 to 1 and measures the level of agreement between multiple ranked variables^15,70^. We calculated Kendall’s *W* between time series for each site within a particular upwelling condition (strong, weak, no upwelling) and determined its significance using Monte Carlo randomizations which shuffled the temperatures within each site randomly^70,71^. The Kendall’s *W* coefficient values reported are corrected for tied ranks and the *p*-values are based on the randomization test (Table 2).

**Table 2:** Kendall’s coefficient of concordance (*W*) measuring the degree of synchrony between sites within a specified upwelling condition. This statistic was measured across sites for the raw time series as well as for scale-averaged wavelet power at annual, monthly and weekly scales. Rows correspond to upwelling condition (strong, weak or no upwelling). Columns correspond to values computed for the raw time series and the resulting *P*-value, and scale averaged power at annual (300–400 days), monthly (15–45 days) and weekly (2–10 days) periodicities, respectively.

	Raw TS	P-value	Annual	P-value	Monthly	P-value	Weekly	P-value
Strong	0.8332439	0.001	0.9178599	0.001	0.7379248	0.001	0.7219196	0.001
Weak	0.8956751	0.001	0.9168151	0.001	0.4209513	0.001	0.3513783	0.001
No	0.9240653	0.001	0.8170147	0.001	0.3998812	0.001	0.4096260	0.001

#### Univariate wavelet analysis

We examined each of the 16 time series via wavelet analysis to determine the relative contribution of each frequency (period) in the signal (time series) to the overall variance of onshore water temperature over time^72,73^. We chose to use wavelet analysis because of its ability to efficiently contend with non-stationarity (i.e., the temporal variation in the statistical properties of a time series), as opposed to more traditional approaches such as spectral analysis, which assumes that the statistical properties of a signal do not vary over time (Fig. 2). This feature of wavelet analysis is particularly critical for analyzing ecological time series, which are often nonlinear and non-stationary^73,74^. Here, we provide a summary of the wavelet analysis method used in this study and include a more detailed description in Appendix S1.

We computed the continuous wavelet transform for each time series using the Morlet wavelet. Using this method allowed us to calculate the wavelet power spectrum, which describes the variability in water temperature occurring at each period or frequency and time point, for each of the 16 time series along the Eastern Atlantic Coast (Fig. 3, rows 2–4). Doing so allowed us to determine the relative contribution of variability at each period or frequency to the total variation observed in each temperature time series. In order to draw comparisons between and within regions characterized by different upwelling indices, the wavelet power spectra were grouped by thermal upwelling index (strong upwelling 1.7–2.7, weak upwelling 0.6–1.0, no upwelling 0.2–0.4). To investigate which scales or periods (1/frequency) dominated the variability of temperature fluctuations across the time series, we calculated the scale-averaged wavelet power for each site^74^. Specifically, we computed the scale-averaged power of the 16 truncated time series for each of the following sets of periodicities: annual [300–400 days], monthly [15–45 days] and weekly [2–10 days] (see Fig. 2c,d). This yielded a time series of average variability in water temperature occurring at each set of periodicities for each site (Fig. 3, rows 2–4). These periodicities were chosen to represent temporal scales of varying ecological relevance. Annual periodicities are representative of a temporal scale often used in climate analyses, whereas monthly and weekly periodicities are representative of temporal scales commonly used in organismal studies, and are particularly informative as a metric of exposure duration, which has been shown to have significant impacts on mortality risk^75^. As before with the raw (daily) time series data, we then calculated Kendall’s *W* to measure the degree of synchrony in temperature variability for each set of periodicities (annual, monthly, weekly) between sites within each upwelling condition. The Kendall’s *W* coefficient values reported are corrected for tied ranks and the *p*-values are based on the randomization test (Table 1). Notably, we refer to synchrony in variability as ‘co-variability’ to distinguish this metric from synchrony in actual temperatures. This distinction is important as two sites can exhibit high co-variability in temperature at the same periodicities but low synchrony because their temperatures peak and trough at different times. This scenario could allow spatial rescue effects to emerge due to the asynchrony of temperature fluctuations across sites^40^.

### Identifying the drivers of spatial patterns

To quantify the potential for spatial thermal refugia, defined here as areas of thermal refuge in space, we calculated the pairwise correlation, coherence and degree of phase locking of onshore water temperatures between sites both within and across upwelling conditions as a function of their geographical distance. A high degree of synchrony in temperature would imply that there are few refugia, as all sites would likely experience periods of extremes at the same time. In contrast, low levels of synchrony in temperature could potentially allow sites to “rescue” one another through the exchange of mobile adults (at high frequencies) or gametes/larvae (at lower frequencies).

#### Raw time series analysis

**Figure 4:**
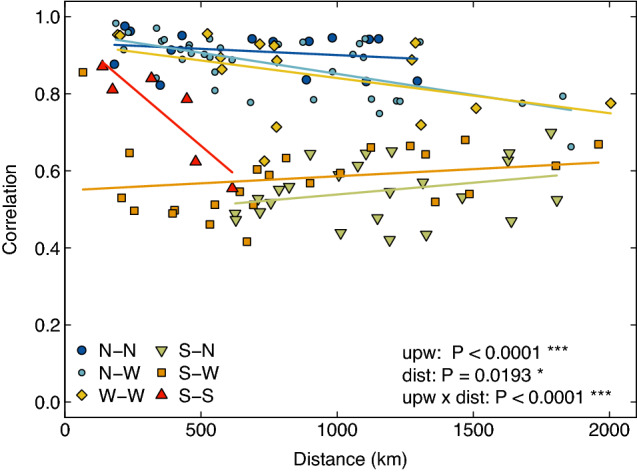
Pairwise correlation in temperature between sites as a function of their geographical distance. Data points are coded by color and shape based on each paired sites’ upwelling condition combination. Strong upwelling pairs (S–S) are represented in red triangles, Strong–Weak upwelling pairs (S–W) are represented in orange squares, Weak upwelling pairs (W–W) are represented in yellow diamonds, No–Weak upwelling pairs (N–W) are represented in small light blue circles, No upwelling pairs (N–N) are represented in large dark blue circles, and Strong-No upwelling pairs (S–N) are represented in green inverted triangles. *P*-values are based on ANCOVA relating average pairwise correlation in temperature between sites to their upwelling condition and geographical distance.

We used Pearson’s product-moment correlation coefficient to measure the strength of the association in onshore water temperature between each pairwise combination of sites (n = 16, 120 pairwise combinations). Pearson’s correlation (*r*) is a parametric statistic that measures the strength of the linear relationship between two datasets, and ranges from − 1 to 1. Additionally, a correlogram was generated to determine how the correlation in water temperature between pairs of sites varied as a function of their geographical distance. A permutation-based ANCOVA was conducted to determine whether (1) the correlation in water temperature between sites decayed with geographical distance (covariate), (2) whether the (adjusted) mean correlation differed between pairs of sites characterized by distinct upwelling conditions (factor), and (3) whether the distance-decay of the correlation differed between sites characterized by distinct upwelling conditions (interaction between factor and covariate; Fig. 4, see tables in Appendix 2).

#### Bivariate wavelet analysis

**Figure 5:**
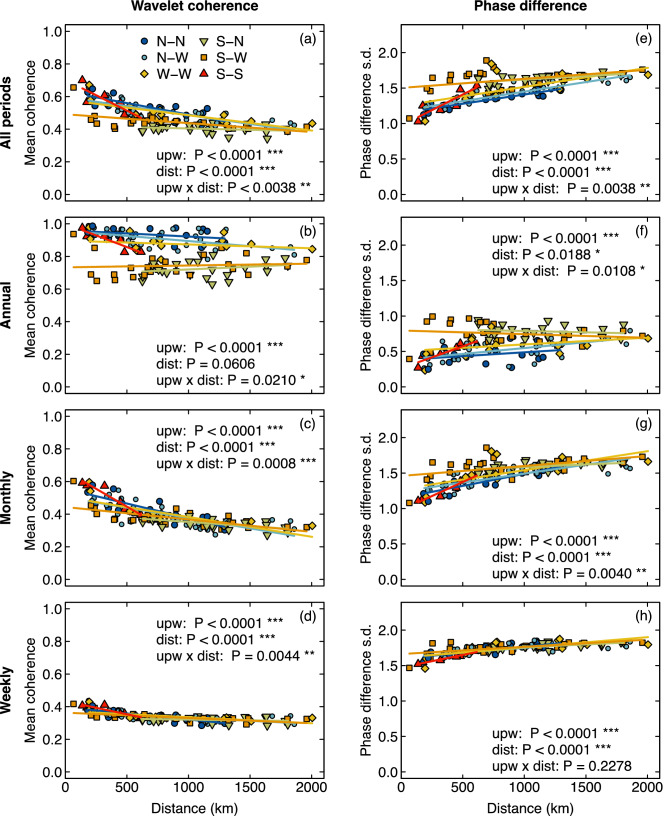
Pairwise wavelet coherence and phase difference in temperature between sites as a function of their geographical distance. Columns represent mean coherence (**a**–**d**) and phase difference spread measured in standard deviations (**e**–**h**). For reference, sites that experience phase synchrony will be characterized by a near zero standard deviation in phase difference. Rows represent all (2–889 days), annual (300–400 days), monthly (15–45 days) and weekly (2–10 day) periods. Data points are coded by color and shape based on each paired sites’ upwelling condition combination. Strong upwelling pairs (S–S) are represented in red triangles, Strong–Weak upwelling pairs (S–W) are represented in orange squares, Weak upwelling pairs (W–W) are represented in yellow diamonds, No–Weak upwelling pairs (N–W) are represented in small light blue circles, No upwelling pairs (N–N) are represented in large dark blue circles, and Strong–No upwelling pairs (S–N) are represented in green inverted triangles. *P*-values are based on ANCOVA relating average pairwise coherence or standard deviation of phase difference in temperature between sites to their upwelling condition and geographical distance.

Due to the general nonlinearity and non-stationarity of ecological time series, we chose to use a bivariate extension of wavelet analysis (wavelet coherence) to assess the temporal variability in the relationship between onshore water temperature and space. Wavelet coherence, which essentially measures the time- and frequency-resolved (squared) cross-correlation between two time series^73,76^, allowed us to examine patterns of time- and frequency-resolved correlation in the variability of water temperature between pairs of sites (Fig. S3.2). We calculated the wavelet coherence for all pairwise combinations of sites within and across upwelling conditons (n = 120 pairwise combinations) in this dataset (Fig. 5). Coherence values range between 0 and 1, with 0 indicating no coherence and 1 indicating strong coherence. Next, we computed the average coherence between each pair of sites across different periodicities (annual [300–400 days], monthly [15–45 days], weekly [2–10 days], as described in Fig. 2b–d and Appendix S3.2) in order to examine the coherence in water temperature variability (or co-variability) between sites at different time scales. These values were plotted as a function of geographical distance (Fig. 5, column 1). Although calculating the mean coherence is useful for determining the degree of cross-correlation of two time series at a particular frequency and time, this metric alone cannot be used to infer the degree to which peaks and troughs in temperature co-occur across sites. To do so, one also needs to quantify the phase difference between time series and determine whether it remains constant. If the difference between the phases of the two time series remains relatively constant over time, then they are considered to be phase locked^73,77^. In order to detect phase locking in water temperature, we calculated the mean and standard deviation of the phase difference for each pairwise combination of sites at each periodicity, as described above and plotted those values against geographical distance (Fig. 5, column 2, Fig. S3.1, columns 2, 3), with low standard deviations indicating phase locking between pairs of time series. We then used permutation-based ANCOVA to determine if the coherence and phase difference of onshore water temperature was driven by upwelling condition (factor), geographical distance (covariate) and/or their interaction (Fig. 4 and see tables in Appendix S2).

## Results

### Identifying the drivers of temporal patterns

#### Raw time series analysis

When the raw time series were grouped based on their thermal upwelling index (e.g., strong upwelling: 1.1–3.0, weak upwelling: 0.5–1.0, no upwelling: 0.0–0.4), we found high levels of synchrony in water temperature across all upwelling conditions (Fig. 3, row 1, Table﻿ 2: *W* = 0.83, 0.89, 0.92 for strong, weak or no upwelling sites, respectively). The high synchrony of onshore water temperature is driven by its seasonal variation, which is characterized by high peaks in Spring and Summer versus low troughs in Fall and Winter, when downwelling events prevail. Despite the strong seasonality of temperature, the effect of upwelling is still visible, as the magnitude of synchrony at each site mirrors its upwelling condition. Specifically, temperature synchrony is lowest at sites characterized by strong upwelling, intermediate at sites characterized by weak upwelling, and strongest at sites characterized by no upwelling (Table 2, Fig. 3). Upwelling thus acts to disrupt the predictable effects of seasonality by reducing temperature synchrony across sites.

#### Univariate wavelet analysis

In computing scale averaged power, the wavelet transform was decomposed into different periodicities of interest (annual [300–400 days], monthly [15–45 days], weekly [2–10 days]) in order to compare the variability of water temperature across different temporal scales (Fig. 3, rows 2–4 respectively). Decomposing the signal at these specific scales revealed that the synchrony of temperature is negatively correlated with its co-variability at ecologically relevant scales. For instance, sites characterized by weak or no upwelling displayed high synchrony in temperature (Table 2, *W* = 0.89, 0.92, respectively) but low co-variability at monthly (Table 2, *W* = 0.42, 0.39, respectively) and weekly scales (Table 2, *W* = 0.35, 0.40, respectively). Additionally, sites characterized by strong upwelling showed lower synchrony in temperature (Table 2, *W* = 0.83), yet high co-variability at monthly (Table 2, *W* = 0.74) and weekly scales (Table 2, *W* = 0.72). The relatively low degree of synchrony in temperature coupled with the high degree of co-variability in regions characterized by strong upwelling suggest that these oceanographic conditions promote the consistent and predictable occurrence of temperature fluctuations whose magnitude and timing differ among sites, particularly at the monthly and weekly scales relevant to organisms (Fig. 3c,d). Upwelling can thus serve as a potent provider of temporal thermal refugia for organisms by preventing the emergence of spatially co-occurring and temporally persistent temperature extremes across sites.

Conversely, in regions characterized by weak or no upwelling, the variation in water temperature is inconsistent across sites. Although sites experience strong fluctuations in the variability of temperature, these fluctuations are only weakly correlated (Table 2, weak/no upwelling monthly, weekly: *W* 0.4; Fig. 3g–l). Hence, the high synchrony of temperature and its low co-variability in regions characterized by either weak or no upwelling suggests that these sites are more likely to suffer from the spatial co-occurrence of longer thermal extremes.

### Identifying the drivers of spatial patterns

#### Raw time series analysis

Plotting the pairwise correlations in the raw water temperature between sites as a function of their geographical distance revealed seasonality as the likely driver of synchrony. Although there was a significant interactive effect of upwelling condition and distance on synchrony (Table S2.1, Fig. 4), we generally observed a high degree of synchrony in temperature between all pairs of sites irrespective of upwelling condition, with fairly weak decays in correlation with distance (Fig. 4). Specifically, for pairs of sites characterized by no upwelling, there was a high degree of correlation in temperature and no decay in that correlation with geographical distance (N–N, *r* > 0.8 correlation over distances spanning > 1300 km). Similarly, correlations in temperature for sites experiencing weak to no upwelling remained high with little to no decay with distance (i.e., W–N and W–W in Fig. 4, *r* = 0.9–0.7 over distances ranging from 250 to 2000 km), a trend likely due to the common seasonal temperature fluctuations experienced by all sites within these regions. While sites belonging to regions characterized by opposite upwelling conditions showed lower correlations in temperature (i.e., S–N in Fig. 4, *r* < 0.6), there was no decay with distance as some sites were dominated by seasonal fluctuations and others by upwelling. These findings based on pairwise correlations in raw temperature time series, combined with the previous analysis of temperature variability, show low co-variability at weekly to monthly scales, suggesting that sites experiencing little to no upwelling do not benefit from temporal refugia. Additionally, the observed high correlations of water temperatures at no/weak sites across 1000 s of km suggests that these sites also cannot serve as spatial refugia. Conversely, the strong decay in correlation with distance for sites characterized by strong upwelling (Fig. 4, S–S, *r* = 0.85–0.5 over 500 km, P = 0.023) suggests that upwelling promotes the emergence of spatial refugia (as temperatures are not correlated over large distances) as well as temporal refugia (high co-variability of temperature).

#### Bivariate wavelet analysis

Overall, regardless of upwelling condition, there was a general reduction in mean pairwise water temperature coherence between sites when the analysis was restricted to monthly and weekly periods (Fig. 5). When mean coherence was calculated across all periods, the signal of upwelling was lost as upwelling currents affect a very narrow band of periodicities in temperature fluctuations (Fig. 5a). However, unlike for the correlations of the raw time series, there is a slight decay in wavelet coherence with geographical distance (Table S2.10 p < 0.001). When only annual periodicities are included, the patterns mirror those observed in the correlation analysis of the raw time series, with all pairs of sites exhibiting high levels of coherence and little to no decay with geographical distance. This is likely because all site time series reflect common seasonal trends due to the limited impact of upwelling on temperature variability occurring at annual to multi-annual periodicities in our relatively short seven-year dataset (Fig. 5b). Average coherence decreased significantly as temporal scale or periodicity decreased (i.e., from annual to weekly), because control shifts from seasonal processes to upwelling (Fig. 5c,d).

To fully characterize the relationship between the temperature of time series from pairs of sites as a function of their geographical distance, we also calculated the mean and the standard deviation of their phase difference. Examining the mean phase difference is useful in determining the average time lag in the fluctuations of water temperature across sites, whereas the standard deviation of phase differences reveals the degree of phase locking over time, with low standard deviation values indicating strong phase locking and high standard deviation values indicating weak to no phase locking. For reference, sites experiencing fully correlated temperature fluctuations would have a value of zero for both the mean and the standard deviation of their phase difference.

Here, regardless of upwelling condition, the mean phase difference was clustered around zero for all periodicities and did not decay with distance (Fig. S3.1, e:h). As expected, there was also a negative correlation between mean coherence and the spread of phase differences across scales (Fig. 5a–d vs. e–h). This trend was most pronounced at annual and weekly scales. For instance, at annual scales, sites in regions with no upwelling exhibited highly coherent (Fig. 5b, R^2^ > 0.9) and in-phase (zero mean phase difference, Fig. S3.1f) temperature fluctuations with low variation in phase difference (low spread; Fig. 5f, 0 < sd < 0.5). Similarly, at weekly scales, the mean coherence and the spread of the phase difference were negatively correlated. Specifically, the mean coherence was low across all sites and declined slightly with distance (Fig. 5d, R^2^ < 0.4, Table S2.5 dist: p < 0.001), whereas the standard deviation of phase difference was high across sites and increased with distance (Fig. 5h, sd > 1.5, Table S2.13 dist: p < 0.001). Overall, there was little to no phase locking at ecologically relevant scales (i.e., monthly and weekly), as evidenced by the spread of the phase differences in the temperature fluctuations being large and increasing with geographical distance (i.e., higher standard deviation of the phase difference). Taken together, the relatively low coherence in water temperature and the low degree of phase locking across 1000 s of km at monthly and weekly temporal scales indicates high spatial heterogeneity in temperature and thus a large potential for spatial rescue across all sites, irrespective of upwelling condition.

## Discussion

Our results indicate that scales at which environmental data are analyzed (and recorded) can have profound effects on the ecological interpretation of the resulting spatiotemporal trends. Many existing predictions about the ecological impacts of increased temperatures are based on spatial or temporal averaging^36,78^. While means can be informative, averaging over space and time can mask important signals (e.g., variability, memory) occurring around the mean^17,29,32,79^. Our results highlight the importance of investigating trends in data resolved in both frequency and time by revealing details about the distribution of spatiotemporal refugia in and around the Canary Current Upwelling system that went undetected when analyzing the raw time series. For instance, analyzing onshore water temperature in both the time and frequency domains revealed information about the co-variability of temperature within (and across) upwelling conditions, illuminating the potential for spatial and temporal rescue effects. Specifically, we found that co-variability of temperature is dependent upon upwelling intensity, with strong upwelling sites showing systematic variation in temperature. Ecologically, networks of sites with a low degree of synchrony in onshore water temperature but a high degree of co-variability (i.e. when water temperatures are changing at similar frequencies but some sites are cooler while others are warmer) are also areas with high potential for temporal refugia. Hence, this shows how the spatial and temporal scales at which data are recorded and analyzed can lead to dramatic differences in ecological responses to climate change.

### The problem of scale in a changing world

Forecasting shifts in the biogeographical patterns of species in response to global change often involves the use of data collected at spatial and temporal scales that are far larger than those directly relevant to organisms^80^. For instance, species range shifts are often classified at macroscales by correlating species habitat or thermal preferences to contemporary or projected climatic variables over large spatial scales^22,36,81–83^. While, these macroscale models result in useful forecasts that can account for many species over large spatial ranges^33,84^, the associated coarse-scale species distribution predictions may also misestimate species’ responses to changing climatic conditions because the proximal drivers of organismal responses to changing weather conditions do not always correspond to those predicted by large-scale climate data^31,85^.

Microclimates and other measures of small-scale environmental heterogeneity are increasingly being recognized as important drivers of ecosystem dynamics^80,84,86^, as species distributions obtained from macroclimate data are not always representative of the range of environmental conditions experienced by organisms in situ^27,86^. Quantifying environmental variation^69,87^ at physiologically^88^ and ecologically^85^ relevant spatiotemporal scales can yield improvements in our understanding of climate-weather-organism interactions^69,89,90^. Our results support these findings. For instance, at coarse temporal scales (annual), onshore water temperature appeared synchronized across all sites regardless of upwelling condition. However, at ecologically relevant temporal scales (monthly and weekly), we found that the synchrony in both temperature and its variability depended on the upwelling condition. This scale-dependent synchronization of the variability of temperature may have important implications for rescue effects. Knowledge about whether or not the variation in temperature is systematic across a region classified by an upwelling condition may improve our ability understand ecological impacts in the context of climate change. For instance, in systems where the variation in temperature is systematic at ecologically relevant temporal scales, we expect that organisms will likely rely primarily on temporal refugia during extreme temperature events. Under this scenario, the synchrony in temperature will be relatively low across sites while the co-variability will be high. Hence, in regions of intense upwelling where sites display relatively low temperature synchrony but high co-variability at monthly and weekly periodicities, organisms are most likely to benefit from local (within-site) temporal rescue effects as temporal refugia will emerge simultaneously during the Summer when high temperature extremes are most deleterious. For example, organisms with sessile adult stages such as *P. vulgata* would benefit from greater local temperature variability in regions of intense upwelling, which would reduce the duration of temperature extremes and thus potentially reduce their mortality rate. In contrast, in regions characterized by weak to no upwelling, temperature synchrony is high but co-variability is low at ecologically relevant time scales (monthly, weekly), and therefore organisms are less likely to systematically experience temporal refugia during temperature extremes. In the absence of temporal refugia, population persistence at these sites would depend more strongly on spatial rescue effects in the form of propagule dispersal from location experiencing more favorable conditions. Pairing these types of analyses with biological data about dispersal potential across different life history stages would be beneficial for ground-truthing the link between spatiotemporal variability in temperature and cryptic thermal refugia.

Additionally, while we found a general negative correlation between mean coherence and the spread of phase differences across periodicities, it is important to note how the temporal scale of the trend affects our interpretation. In considering only annual scales, one might predict a low potential for spatial rescue effects as water temperature appears to be spatially homogenous with sites both in phase and nearly phase locked. However, the temperatures that organisms experience and which drive their physiology and ecology cannot be quantified by trends at annual scales. Thus, analyzing onshore water temperature resolved in both time and frequency at ecologically relevant scales revealed hidden spatial rescue effects for sites experiencing weak to no upwelling that were masked when the analysis was not resolved in the frequency domain. In sum, these analyses illuminated high potential for spatial rescue effects across all upwelling conditions, with sites characterized by high upwelling also benefiting from temporal refugia. The novel insights that we gained by using fine-scale environmental data and decomposing its variability in the time and frequency domains demonstrate the potential for this framework to be applied to other systems in order to forecast and manage species persistence under different climate scenarios.

### Environmental refugia and rescue effects

Identifying ecological refugia is an emerging strategy used to predict species’ capacity to cope with climatic change^84,91^. In natural systems, which are environmentally heterogeneous, populations of organisms that cannot acclimate will rely on spatial or temporal refugia to persist. Historically, glacial and interglacial periods have provided a useful record from which to gain insights on range contractions and expansions and define refuges for flora and fauna at the level of species^92,93^. Expanding from this work, ecological refugia came to be defined as areas that facilitate the persistence of species in the face of climatic change. To this end, recent studies have largely classified refugia by habitat^33,65^ or climatic stability^41,94^. One common hypothesis derived from this body of work postulates that species distributions will shift poleward as temperatures increase. While intuitive and appealing as a heuristic simplification, the data have not supported this hypothesis for a wide variety of species^36^. This is likely due to the fact that many projections of range shifts refer exclusively to those expected in response to changes in the mean of the climatic variable of interest, which may not always be reflective of the underlying frequencies (weather) driving survival and reproduction^85^. Results from our study suggest that identifying areas with potential for spatiotemporal rescue by understanding how climatic variables will vary in space and time can provide insights as to where populations are more likely to persist even when they seemingly cannot ‘keep pace’ with climatic shifts. While here we focus on water temperature, the approach for which we advocate can be applied to other environmental variables known to vary at high frequencies and to drive ecological responses, such as ocean pH^95^.

Eastern Boundary Upwelling Systems are both ecologically and economically important regions which are projected to change under shifting climate conditions^60,96,97^. These systems have a high degree of spatial heterogeneity in temperature, as sites throughout each region experience a gradient of upwelling intensities. Furthermore, these coastal upwelling systems often exhibit cooler surface water temperatures than their offshore counterparts^96,98^. This has prompted recent work which highlights the potential for upwelling zones to serve as marine climate and thermal refugia^41^. A deeper understanding of the associated factors that both promote and erode the emergence of refugia in upwelling regions can benefit management and conservation planning.

Theory suggests that the loss of spatiotemporal heterogeneity due to the synchronization of environmental fluctuations can promote the spatial synchrony of population dynamics and thus increase extinction risk across scales by preventing the emergence of spatial rescue effects^99^. In contrast, under low spatial synchrony, local populations at high abundances can rescue those at low abundances (e.g., after a mortality event), and thus reduce extinction risk at both local and regional scales^38,39,100^. In this study, by examining how the cryptic variability of onshore water temperature changed across space within an upwelling system, we found that the strong spatial heterogeneity in water temperature led to a low degree of phase locking between sites, indicating a high potential for spatial refugia via rescue effects across the upwelling system. This spatial refugia in onshore water temperature can be crucial for the persistence of intertidal species. Sessile or slow-moving organisms are limited in their ability to behaviorally thermoregulate during temperature extremes (e.g., by moving to deeper water), and moreover, as broadcast spawners, the variability of water temperature has important consequences for the survival and recruitment of propagules. For example, at their meridional distributional limit in Southern Europe, low spatial synchrony in temperature conditions between sites (low degree of phase locking) may be an important mechanism underpinning the source-sink dynamics necessary for recruitment success and overall persistence of metapopulations of cold-water species such as the brown algae *Ascophyllum nodosum*, *Laminaria hyperborea*, *Himanthalia elongata*, or the limpet *Patella vulgata*.

We found that locations characterized by a high thermal upwelling index also had a high potential to function as temporal refugia. Temporal refugia may be crucial for organisms in locations that experience a high degree of spatial synchrony in climatic conditions. In this scenario, organisms are likely to rely on local temporal refugia which may result in increased clustering of habitat patches within-sites, another mechanism thought to promote the persistence of metapopulations^101^. Specifically, quantifying spatiotemporal refugia in terms of synchrony as was done in this study allows us to glean potential for persistence regardless of the environmental variable or system in question. Here, spatiotemporal refugia can promote organismal persistence by reducing both the magnitude and the duration of extreme temperatures at local sites characterized by strong upwelling or decreasing their co-occurrence in space and time across the region. Overall, the ability to reveal cryptic spatiotemporal refugia highlights the power of this approach in an era of global change where climatic uncertainty will be the new normal. Understanding the variability of climatic variables can provide us with a powerful tool for tackling environmental uncertainty and documenting areas of refuge thereby extending other more static approaches.

Although there has been a concerted effort to forecast how mean conditions will change under different greenhouse gas emission scenarios, the potential for shifts in the spatiotemporal variability of environmental conditions over the coming decades has garnered less attention. For instance, while Seabra et al.^52^ found that the mean sea surface temperature in Eastern Boundary Upwelling Systems has not increased at the same rate as temperature outside these regions, comparisons of the variability of temperature across upwelling and non-upwelling systems remain scarce. Given that we found that the variability of temperature can be informative for elucidating ecologically relevant refugia, an important next step would be to determine how that variability differs across upwelling systems and neighboring regions. In fact, knowing more about the variability of temperature in areas outside of Eastern Boundary Upwelling Systems, which are warming faster^54^, would enable the identification of areas with high or low potential for spatial or temporal refugia due to high synchronization of variability. This will be particularly important moving forward as recent work has identified poleward shifts of EBUS for the twenty-first century^60,102,103^, which has the potential to affect areas previously outside of EBUS that are likely to begin to exhibit different oceanographic characteristics in the wake of these shifts.

Exploring spatiotemporal patterns in other environmental and oceanographic variables will be an important next step. For instance, while upwelling can lead to lower temperatures and higher nutrient levels, upwelling regions also experience lower oxygen saturation^96^. Globally, increased temperatures are expected to intensify hypoxic conditions^104,105^. Thus, a natural extension of this work would be to examine variation in other ecologically important environmental variables in both the time and frequency domains (e.g., oxygen concentration, carbonate chemistry). It is also important to note that since upwelling systems are changing at different rates^60,106^, it will be necessary to quantify region-specific variation in the environment (e.g., temperature, pH, oxygen) and avoid generalizing across locations that experience ‘similar’ upwelling intensities (e.g., sites with high upwelling indices in Canary Current System vs California Current System). Quantifying general climatic trends (via computing global averages and climate velocity trajectories) has resulted in important baseline predictions of how species ranges are likely to shift in space and time, and an increased understanding of the mechanisms driving these patterns will improve our ability to understand changes in community composition and species assemblages. Since temperature extremes are often key drivers of population dynamics^107,108^ and are becoming increasingly common^109,110^, it is important to adopt statistical approaches that are able to decompose temperature variability at ecologically relevant scales in order to gain a more holistic interpretation of cryptic temporal and spatial refugia and their potential influence on the distribution of species.

## Supplementary Information

Supplementary Information 1.

Supplementary Information 2.

## Data Availability

The data that support the findings of this study are available from FL and RS upon reasonable request.
