# Supplementary material for: Coastal upwelling generates cryptic temperature refugia
Source: Sci Rep. 2022 Nov 11;12:19313. doi: 10.1038/s41598-022-23717-5 (PMC9652353; doi:10.1038/s41598-022-23717-5)

Supplementary Information

Appendix S1: Wavelet analysis

Background

The application of spectral analysis to ecological and environmental time series can provide invaluable insights about the dynamics of ecosystems as well as their drivers (Benincà et al. 2009, Wootton and Forester 2013). Spectral analysis, often performed via the Fourier transform, decomposes a time series commonly referred to as a signal into a set of sinusoidal waves each characterized by a different frequency or 1/period and amplitude or power (Chatfield 1996, Cazelles et al. 2008). This decomposition can thus be used to determine the relative contribution of the variability observed at each frequency to the total variation in the time series. Unfortunately, because spectral analysis is not time-resolved and thus assumes that a time series is largely stationary (i.e., its statistical properties are constant), it cannot be used to understand how the relative contribution of variability observed at each frequency changes over time. Although windowed spectral analysis can overcome some of these limitations, this approach is generally inefficient and can lead to information loss when applied to non-stationary ecological and environmental time series (Benincà et al. 2008, Cazelles et al. 2008, Rouyer et al. 2008, Gouhier et al. 2010). Wavelet analysis is ideal for efficiently extracting information from such non-stationary time series because it is both time- and frequency-resolved. Here, we used wavelet analysis to determine how fluctuations in onshore water temperature varied within and across sites at 16 locations along the European Atlantic Coast. Below, we provide a summary of the univariate and bivariate wavelet methods used to produce the results presented in the main text. More detailed descriptions of wavelet analysis can be found elsewhere in the literature (e.g., Torrence and Compo 1998, Grinsted et al. 2004, Cazelles et al. 2008, Iles et al. 2012). All of our analyses were conducted using the biwavelet package for R written by T.C. Gouhier.

Wavelet analysis

Wavelet analysis is able to resolve both the time and frequency domains of a signal, via the wavelet transform. Specifically, a transforming function (mother wavelet) is passed through a signal via windows τ across a series of scales s. For our study of water temperature time series, we chose to use the complex Morlet wavelet (a commonly used wavelet), which represents a sine wave modulated by a Gaussian function (Fig. S1-2; Torrence and Compo 1998):

$$\psi_0(t) = \pi^{-1/4} e^{i\omega_0 t} e^{-t^2/2}$$

Where i is the imaginary unit, t represents nondimensional time, and $\omega_0 = 6$ is the nondimensional frequency (Torrence and Compo 1998). The continuous wavelet transform of a discrete time series $x(t)$ with equal spacing δt and length T is defined as the convolution of $x(t)$ with a normalized Morlet wavelet (Torrence and Compo 1998, Grinsted et al. 2004):

$$W_x(s,\tau) = \sqrt{\frac{\delta t}{s}\sum_{t=0}^{T-1} x(t)\psi_0} * \left(\frac{(t-\tau)\delta t}{s}\right)$$

where * indicates the complex conjugate. By varying the wavelet scale s (i.e., dilating and contracting the wavelet) and translating it along localized time position τ, one can calculate the wavelet coefficients $W_x(s,\tau)$ across the different scales s and time positions τ. These wavelet coefficients can be used to compute the bias-corrected local wavelet power, which describes how the contribution of each frequency or period in the time series varies in time (Torrence and Compo 1998, Liu et al. 2007, Cazelles et al. 2008):

$$W_x^2(s,\tau) = 2^s |W_x(s,\tau)|^2$$

Where 2^s is the bias correction factor (Liu et al. 2007). The local wavelet power spectrum can then be visualized via contour plots and heatmaps (Grinsted et al. 2004, Cazelles et al. 2008).

The scale s of the Morlet wavelet is related to the Fourier frequency f (Maraun and Kurths 2004, Cazelles et al. 2008):

$$\frac{1}{f} = \frac{4\pi s}{\omega_0 + \sqrt{2 + \omega_0^2}}$$

When $\omega_0 = 6$, the scale s is approximately equal to the reciprocal of the Fourier frequency f such that $s \approx \frac{1}{f}$. Hence, the scale can be converted to the Fourier frequency $f \approx \frac{1}{s}$ or period $p = \frac{1}{f} \approx s$.

Zero-padding and the cone of influence

While the continuous wavelet transform can be approximated by using discrete Fourier transforms to compute *T* convolutions for each scale s, it is more efficient to use discrete Fourier transforms to calculate all *T* convolutions simultaneously (Torrence and Compo 1998). However, this method will introduce errors in the estimation of the local wavelet power spectrum at both the beginning and the end of a finite time series because the Fourier transform assumes that the data is periodic (Torrence and Compo 1998, Cazelles et al. 2008). A workaround for this issue is to pad the time series with zeros before the wavelet transform is computed. Typically, enough zeros are added to both ends of the time series so that its length *T* is a power of two in order to take advantage of the computational efficiencies afforded by the Fast Fourier Transform (Torrence and Compo 1998, Cazelles et al. 2008). Since this procedure reduces the reliability of the estimation of the local wavelet spectrum by a factor of e^{-2}, the region where zero padding occurred is termed the 'cone of influence' (COI). The areas below the COI are often shaded in figures in order to highlight the estimation issues associated with zero padding and the potential impact of edge effects on the interpretation of the results (Torrence and Compo 1998, Cazelles et al. 2008).

Statistical significance testing

The wavelet power spectra associated with the observed temperature time series were compared to those expected based on a null model in order to determine their statistical significance. Because most ecological and environmental time series tend to exhibit strong temporal autocorrelation (i.e., high power associated with low frequencies; e.g. Beninca et al. 2009, see Ruokolainen et al. 2009 for review), we generated temporally autocorrelated time series based on a first order autoregressive process [AR(1)] to serve as our null model. The power spectrum $p(k)$ of our null model was then calculated as (Gilman et al. 1963):

$$p(k) = \frac{1 - \alpha^2}{1 + \alpha^2 - 2\alpha\cos(2\pi k\,/N)}$$

where the autocorrelation coefficient α at time lag 1 was estimated from the observed time series and $k = 0, \dots, \frac{N}{2}$ represents the frequency index. The distribution of the local wavelet power spectrum $|W_x(s,\tau)|^2$ of the null model at scale s and time position τ is then (Torrence and Compo 1998):

$$|W_x(s,\tau)|^2 \sim \frac{\sigma^2}{2} p(k)\mathcal{X}_2^2$$

Where k represents the frequency index, σ^2 represents the variance of the time series and $\mathcal{X}_2^2$ represents the $\mathcal{X}^2$ distribution with 2 degrees of freedom. The value of $p(k)$ is the mean wavelet power spectrum at frequency k that corresponds to the wavelet scale s (Torrence and Compo 1998). Using this equation, one can construct 95% confidence contour lines at each scale using the 95th percentile of the $\mathcal{X}_2^2$ distribution (Torrence and Compo 1998). Within these contoured regions, the wavelet power spectrum associated with the observed temperature time series differs from that expected under the null model at the $\alpha = 0.05$ significance level.

Wavelet coherence

Wavelet coherence quantifies the strength of the covariation between two time series across different scales or frequencies and times. Wavelet coherence thus represents the frequency- and time-resolved correlation between two time series (Cazelles et al. 2008). To compute wavelet coherence, one must first take the wavelet transforms $W_x(s,\tau)$ and $W_y(s,\tau)$ of time series $x(t)$ and $y(t)$, respectively, and then calculate the cross-wavelet (essentially the frequency- and time-resolved covariance) via the following equation (Torrence and Compo 1998, Grinsted et al. 2004):

$$W_{x,y}(s,\tau) = W_x(s,\tau)W_y^*(s,\tau)$$

where * indicates complex conjugation. The wavelet coherence is then defined as:

$$R_{x,y}^2(s,\tau) = \frac{\left|\langle s^{-1}W_{x,y}(s,\tau)\rangle\right|^2}{\langle s^{-1}|W_x(s,\tau)|^2\rangle\langle s^{-1}\left|W_y(s,\tau)\right|^2\rangle}$$

Where $\langle\cdot\rangle$ denotes smoothing in both time τ and scale s and $0 \leq R_{x,y}^2(s,\tau) \leq 1$. Here, the time smoothing is done via a filter derived from the absolute value of the wavelet function at each scale, normalized to have a total weight of unity, which is a Gaussian function $e^{\frac{-t^2}{2s^2}}$ for the Morlet wavelet. The scale smoothing is done with a boxcar function of width 0.6, which corresponds to the decorrelation scale of the Morlet wavelet (Torrence and Compo 1998, Grinsted et al. 2004).

Since we used the complex Morlet wavelet, we can compute $\phi_{x,y}(s,\tau)$, which describes the local phase difference between the fluctuations in the time series $x(t)$ and $y(t)$ at each scale s and time location τ (Torrence and Webster 1998):

$$\phi_{x,y}(s,\tau) = \tan^{-1}\left(\frac{\Im\left(\langle s^{-1}W_{x,y}(s,\tau)\rangle\right)}{\Re\left(\langle s^{-1}W_{x,y}(s,\tau)\rangle\right)}\right)$$

The phase difference $\phi_{x,y}(s,\tau)$ can be represented with arrows that indicate whether the time series are moving in the same direction (i.e., in phase: arrows pointing to the right indicating a 0 degree/radian difference in the phases), in opposite directions (i.e., in anti-phase: arrows pointing to the left indicating a 180 degree or π radian difference in the phases), or some intermediate scenario.

References

Benincà, E., J. Huisman, R. Heerkloss, K. D. Jöhnk, P. Branco, E. H. Van Nes, M. Scheffer, and S. P. Ellner. 2008. Chaos in a long-term experiment with a plankton community. Nature 451:822–825.

Benincà, E., K. D. Jöhnk, R. Heerkloss, and J. Huisman. 2009. Coupled predator–prey oscillations in a chaotic food web. Ecology letters 12:1367–1378.

Beninca, E., K. D. Johnk, R. Heerkloss, and J. Huisman. 2009. Coupled predator-prey oscillations in a chaotic food web. Ecology Letters 12:1–12.

Cazelles, B., M. Chavez, D. Berteaux, F. Ménard, J. O. Vik, S. Jenouvrier, and N. C. Stenseth. 2008. Wavelet Analysis of Ecological Time Series. Oecologia 156:287–304.

Chatfield, C. 1996. The analysis of time series: an introduction. Fifth edition. Chapman & Hall.

Gilman, D. L., F. J. Fuglister, and J. M. Mitchell. 1963. On the Power Spectrum of "Red Noise." Journal of the Atmospheric Sciences 20:182–184.

Gouhier, T. C., F. Guichard, and B. A. Menge. 2010. Ecological processes can synchronize marine population dynamics over continental scales. Proceedings of the National Academy of Sciences 107:8281–8286.

Grinsted, A., J. C. Moore, and S. Jevrejeva. 2004. Application of the cross wavelet transform and wavelet coherence to geophysical time series. Nonlinear Processes in Geophysics 11:561–566.

Iles, A. C., T. C. Gouhier, B. A. Menge, J. S. Stewart, A. J. Haupt, and M. C. Lynch. 2012. Climate-driven trends and ecological implications of event-scale upwelling in the California Current System. Global Change Biology 18:783–796.

Liu, Y., X. San Liang, and R. H. Weisberg. 2007. Rectification of the Bias in the Wavelet Power Spectrum. Journal of Atmospheric and Oceanic Technology 24:2093–2102.

Maraun, D., and J. Kurths. 2004. Cross wavelet analysis: significance testing and pitfalls. Nonlinear Processes in Geophysics 11:505–514.

Rouyer, T., J.-M. Fromentin, F. Ménard, B. Cazelles, K. Briand, R. Pianet, B. Planque, and N. C. Stenseth. 2008. Complex interplays among population dynamics, environmental forcing, and exploitation in fisheries. Proceedings of the National Academy of Sciences 105:5420–5425.

Ruokolainen, L., A. Lindén, V. Kaitala, and M. S. Fowler. 2009. Ecological and evolutionary dynamics under coloured environmental variation. Trends in Ecology & Evolution 24:555–563.

Torrence, C., and G. P. Compo. 1998. A Practical Guide to Wavelet Analysis. Bulletin of the American Meteorological Society 79:61–78.

Torrence, C., and P. J. Webster. 1998. The annual cycle of persistence in the El Niño/Southern Oscillation. Quarterly Journal of the Royal Meteorological Society 124:1985–2004.

Wootton, J. T., and J. D. Forester. 2013. Complex Population Dynamics in Mussels Arising from Density-Linked Stochasticity. PloS one 8:e75700.

Appendix S2: Permutation based ANCOVA tables

Raw Time Series: Correlation

Table S2.1 Results of permutation-based ANCOVA testing the effects of upwelling regime and distance on the pairwise correlation of temperature for 16 along the Canary Current System. Upwelling regimes range from strong upwelling to no upwelling. P-values from permutation test (P_{perm}) are reported for each factor in our analysis.

Effect	DF	F	P	P_{perm}
Upwelling	5	108.99	< 0.0001	0.001
Distance	1	8.69	0.0193	0.018
Upw x Dist	5	8.32	< 0.0001	0.001

Wavelet Coherence: Mean coherence

Table S2.2. Results of permutation-based ANCOVA testing the effects of upwelling regime and distance on the mean coherence across all periodicities. Upwelling regimes range from strong upwelling to no upwelling. P-values from permutation test (P_{perm}) are reported for each factor in our analysis.

Effect	DF	F	P	P_{perm}
Upwelling	5	46.46	< 0.0001	0.001
Distance	1	94.68	< 0.0001	0.001
Upw x Dist	5	7.29	0.0038	0.001

Table S2.3. Results of permutation-based ANCOVA testing the effects of upwelling regime and distance on the mean coherence at annual periods.

Effect	DF	F	P	P_{perm}
Upwelling	5	71.17	< 0.0001	0.001
Distance	1	3.08	0.0606	0.059
Upw x Dist	5	4.42	0.0210	0.007

Table S2.4. Results of permutation-based ANCOVA testing the effects of upwelling regime and distance on the mean coherence at monthly periods.

Effect	DF	F	P	P_{perm}
Upwelling	5	18.30	< 0.0001	0.001
Distance	1	145.14	< 0.0001	0.001
Upw x Dist	5	4.54	0.0008	0.002

Table S2.5. Results of permutation-based ANCOVA testing the effects of upwelling regime and distance on the mean coherence at weekly periods.

Effect	DF	F	P	P_{perm}
Upwelling	5	11.07	< 0.0001	0.001
Distance	1	57.91	< 0.0001	0.001
Upw x Dist	5	4.82	0.0044	0.008

Wavelet Coherence: Mean phase difference

Table S2.6. Results of permutation-based ANCOVA testing the effects of upwelling regime and distance on the mean phase difference of coherence across all periodicities. Upwelling regimes range from strong upwelling to no upwelling. P-values from permutation test (P_{perm}) are reported for each factor in our analysis.

Effect	DF	F	P	P_{perm}
Upwelling	5	8.08	0.5514	0.547
Distance	1	0.12	0.0566	0.060
Upw x Dist	5	2.24	0.9304	0.935

Table S2.7. Results of permutation-based ANCOVA testing the effects of upwelling regime and distance on the mean phase difference of coherence at annual periods.

Effect	DF	F	P	P_{perm}
Upwelling	5	10.27	0.0004	0.002
Distance	1	2.15	0.6490	0.676
Upw x Dist	5	0.77	0.3948	0.403

Table S2.8. Results of permutation-based ANCOVA testing the effects of upwelling regime and distance on mean phase difference of coherence at monthly periods.

Effect	DF	F	P	P_{perm}
Upwelling	5	11.32	0.1564	0.155
Distance	1	14.46	0.2439	0.255
Upw x Dist	5	2.48	0.8408	0.845

Table S2.9. Results of permutation-based ANCOVA testing the effects of upwelling regime and distance on the mean phase difference of coherence at weekly periods.

Effect	DF	F	P	P_{perm}
Upwelling	5	6.04	0.2130	0.206
Distance	1	16.35	0.8481	0.856
Upw x Dist	5	3.62	0.5687	0.561

Wavelet Coherence: Standard deviation of phase difference

Table S2.10. Results of permutation-based ANCOVA testing the effects of upwelling regime and distance on the standard deviation of phase difference of coherence across all periodicities. Upwelling regimes range from strong upwelling to no upwelling. P-values from permutation test (P_{perm}) are reported for each factor in our analysis.

Effect	DF	F	P	P_{perm}
Upwelling	5	42.36	< 0.0001	0.001
Distance	1	115.63	< 0.0001	0.001
Upw x Dist	5	5.30	0.0038	0.003

Table S2.11. Results of permutation-based ANCOVA testing the effects of upwelling regime and distance on the standard deviation of phase difference of coherence at annual periods.

Effect	DF	F	P	P_{perm}
Upwelling	5	27.86	< 0.0001	0.001
Distance	1	9.03	0.0188	0.014
Upw x Dist	5	3.77	0.0108	0.004

Table S2.12. Results of permutation-based ANCOVA testing the effects of upwelling regime and distance on the standard deviation of phase difference of coherence at monthly periods.

Effect	DF	F	P	P_{perm}
Upwelling	5	21.65	< 0.0001	0.001
Distance	1	121.79	< 0.0001	0.001
Upw x Dist	5	4.81	0.0040	0.004

Table S2.13. Results of permutation-based ANCOVA testing the effects of upwelling regime and distance on the standard deviation of phase difference of coherence at weekly periods.

Effect	DF	F	P	P_{perm}
Upwelling	5	14.26	< 0.0001	0.001
Distance	1	87.50	< 0.0001	0.001
Upw x Dist	5	3.14	0.2278	0.222

Appendix S3: Evidence for synchrony between sites

Figure S3.1. Coherence and phase difference as a function of geographical distance between sites. Columns represent mean coherence (a-d), mean phase difference (e-h) and standard deviation of phase difference (i-l). For reference, sites that experience full-synchrony of water temperature would have a value close to zero for the both the mean and the standard deviation of phase difference. Rows represent all (2-889 days), annual (300-400 days), monthly (15-45 days) and weekly (2-10 day) periods. Data points are coded by color and shape based on each paired sites' upwelling regime combination. Strong upwelling pairs (S-S) are represented in red triangles, Strong-Weak upwelling pairs (S-W) are represented in orange squares, Weak upwelling pairs (W-W) are represented in yellow diamonds, No-Weak upwelling pairs (N-W) are represented in small light blue circles, No upwelling pairs (N-N) are represented in large dark blue circles, and Strong-No upwelling pairs (S-N) are represented in green inverted triangles. *P*-values are based on ANCOVA relating average pairwise coherence, mean of phase difference, or standard deviation of phase difference in temperature between sites to their upwelling regime and geographical distance.

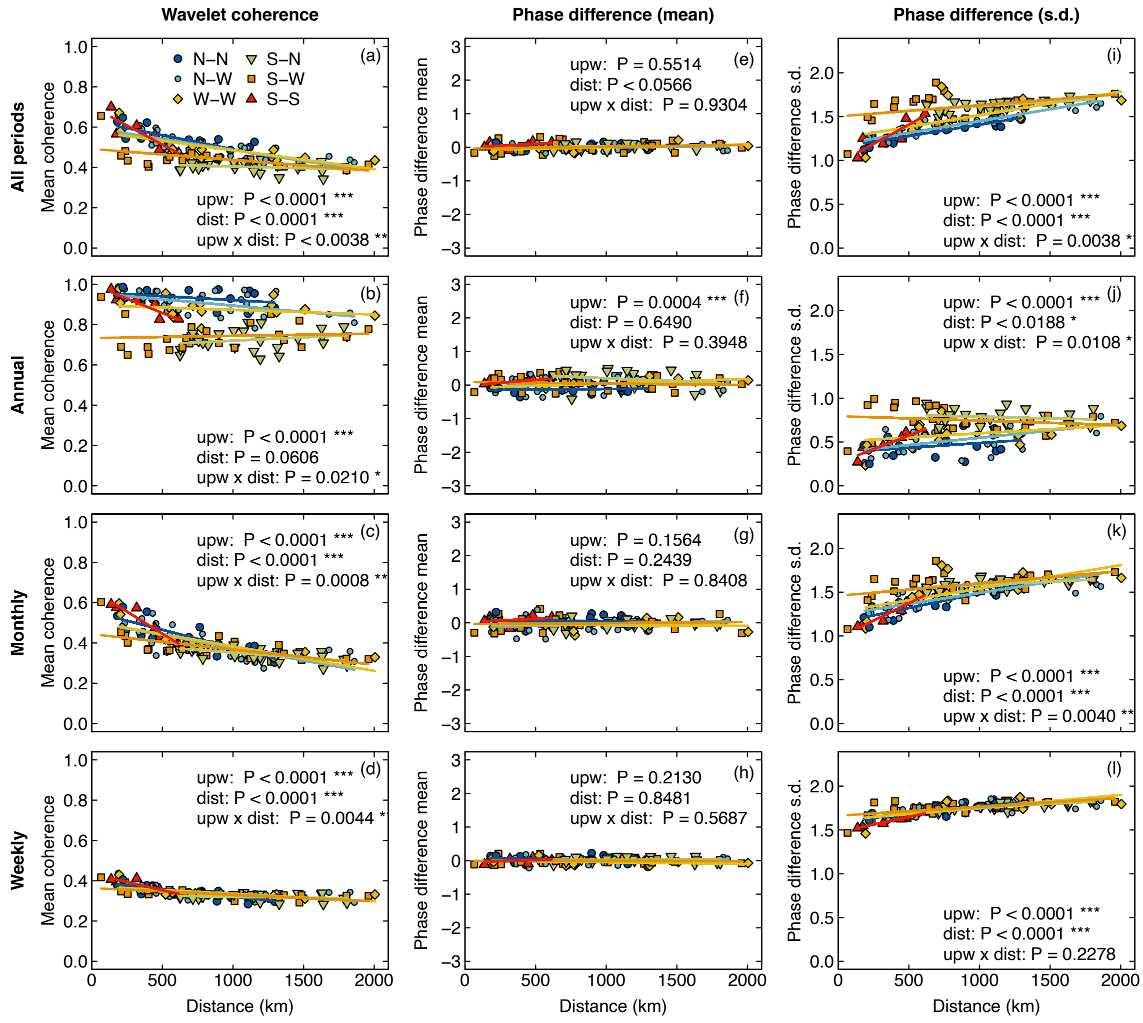

Figure S3.2. Coherence wavelet analyses indicating the relationship in variability of onshore water temperature between (a) sites characterized by similar upwelling regimes (S-S) and (b) sites characterized by opposing regimes (S-N). Color indicates wavelet coherence ranging from high (warm colors) to low (cool colors). Right pointing arrows indicate in phase dynamics, left pointing arrows indicate antiphase dynamics. The white-shaded region represents estimates of wavelet coherence that are potentially affected by edge effects. Black contours designate regions of significantly high temporal covariation compared to a null model consisting of two independent red noise signals.

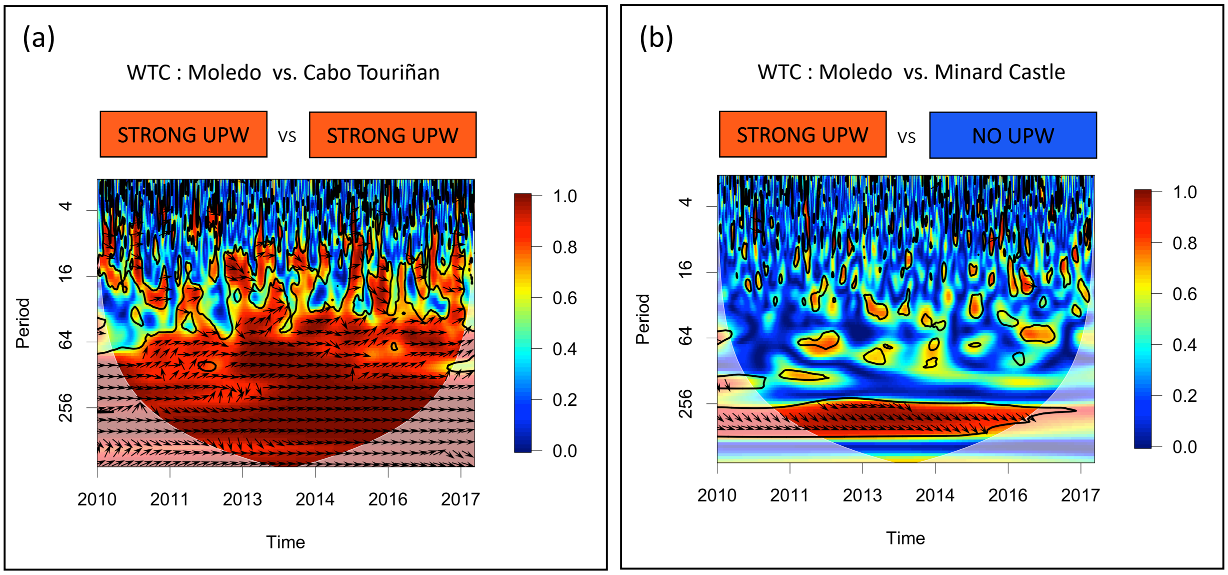

Supplement: Supplementary file 1 — Supplementary Information 1. [file 41598_2022_23717_MOESM1_ESM.pdf]
