# Supplementary material for: Coastal upwelling generates cryptic temperature refugia
Source: Sci Rep. 2022 Nov 11;12:19313. doi: 10.1038/s41598-022-23717-5 (PMC9652353; doi:10.1038/s41598-022-23717-5)
Supplement: Supplementary file 2 — Supplementary Information 2. [file 41598_2022_23717_MOESM2_ESM.pdf]

Appendix S4: Sensitivity analysis for upwelling classification

To determine the robustness of the classification scheme based solely on each site’s Thermal Upwelling Index (no upwelling: 0.0-0.4, weak upwelling: 0.5-1.0, strong upwelling: 1.1-3.0, see Fig. 1 and Table 1 in main text), we ran our analysis on an alternate classification scheme. Specifically, we used independent oceanographic information about the geographical distribution of upwelling in the region to reclassify three sites in Scotland and England from ‘weak upwelling’ to ‘no upwelling’ (sites ‘A’ [South Cairn, Scotland], ‘E’ [E. Wembury, England], and ‘F’ [Landunvez, France]). All analyses described in the main text were then repeated using this new classification scheme, which yielded the results included in Appendix S4. Overall, the new classification scheme had no qualitative effect and only a very small quantitative effect on the results (see Table S4.1 and Fig S4.1-4.4). Hence, our results are robust to alternative classification schemes that are not fully based on each site’s Thermal Upwelling Index.

Table S4.1. Kendall's coefficient of concordance (*W*) measuring the degree of synchrony between sites within a specified upwelling condition. This statistic was measured across sites for the raw time series as well as for scale-averaged wavelet power at annual, monthly and weekly scales. Rows correspond to upwelling condition (strong, weak or no upwelling). Columns correspond to values computed for the raw time series and the resulting *P*-value, and scale averaged power at annual (300-400 days), monthly (15-45 days) and weekly (2-10 days) periodicities, respectively.

	Raw TS	P-value	Annual	P-value	Monthly	P-value	Weekly	P-value
Strong	0.8332439	0.001	0.9178599	0.001	0.7379248	0.001	0.7219196	0.001
Weak	0.8810938	0.001	0.9237022	0.001	0.7703277	0.001	0.7317397	0.001
No	0.9190720	0.001	0.8612175	0.001	0.3924525	0.001	0.3278119	0.001

Figure S4.1. Time series and scale-averaged wavelet power for daily onshore water temperature at all sites. Columns represent site groupings based on upwelling strength (i.e., strong upwelling, weak upwelling, no upwelling). The first row depicts the raw time series, and subsequent rows represent scale-averaged power at annual (300-400 days), monthly (15-45 days) and weekly (2-10 days) periodicities, respectively. Time series for individual sites are represented by grey lines and bold black lines depict the mean time series after averaged across all sites. The background color indicates Spring/Summer months (orange) or Fall/Winter months (blue).

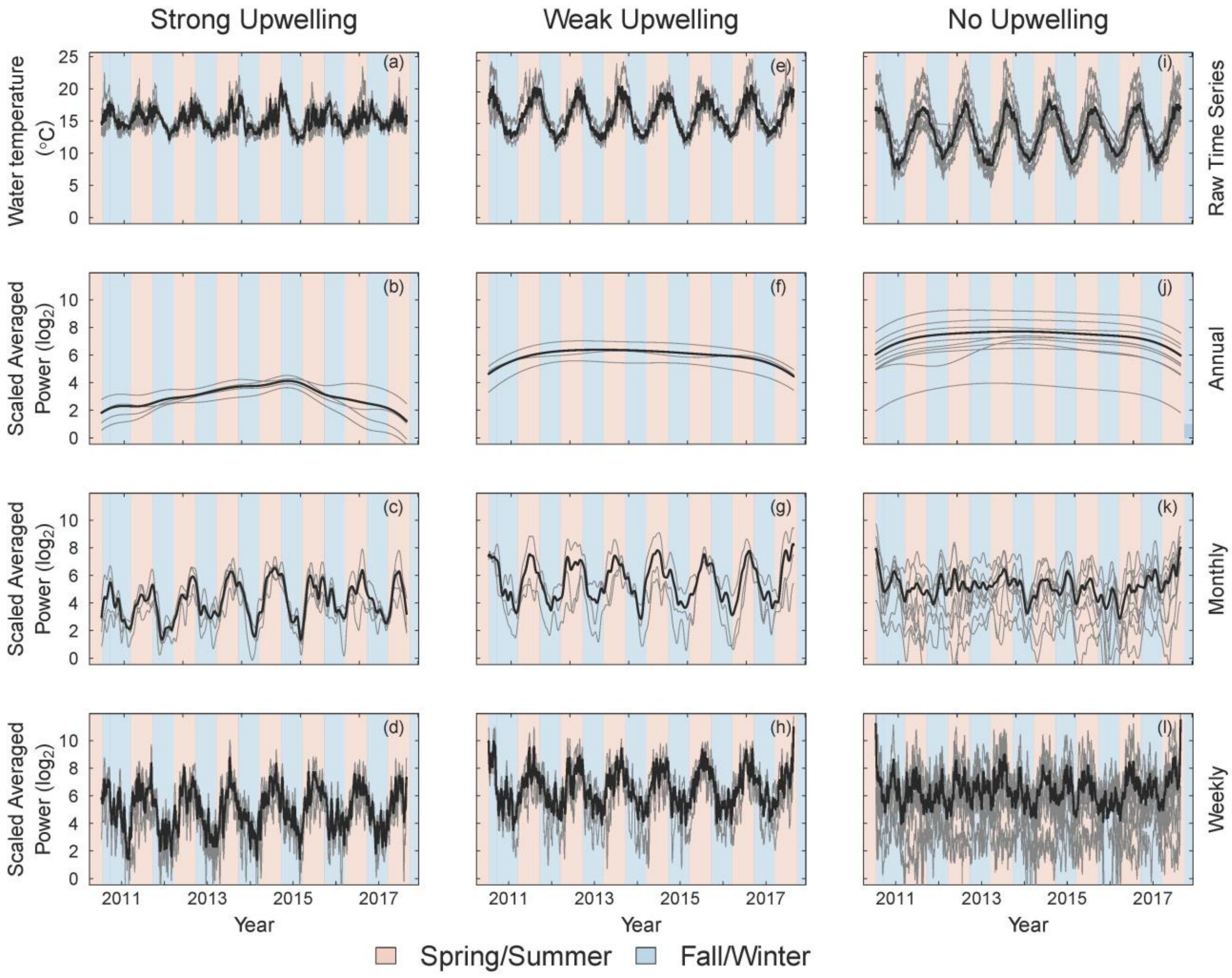

Figure S4.2. Pairwise correlation in temperature between sites as a function of their geographical distance. Data points are coded by color and shape based on each paired sites' upwelling condition combination. Strong upwelling pairs (S-S) are represented in red triangles, Strong-Weak upwelling pairs (S-W) are represented in orange squares, Weak upwelling pairs (W-W) are represented in yellow diamonds, No-Weak upwelling pairs (N-W) are represented in small light blue circles, No upwelling pairs (N-N) are represented in large dark blue circles, and Strong-No upwelling pairs (S-N) are represented in green inverted triangles. *P*-values are based on ANCOVA relating average pairwise correlation in temperature between sites to their upwelling condition and geographical distance.

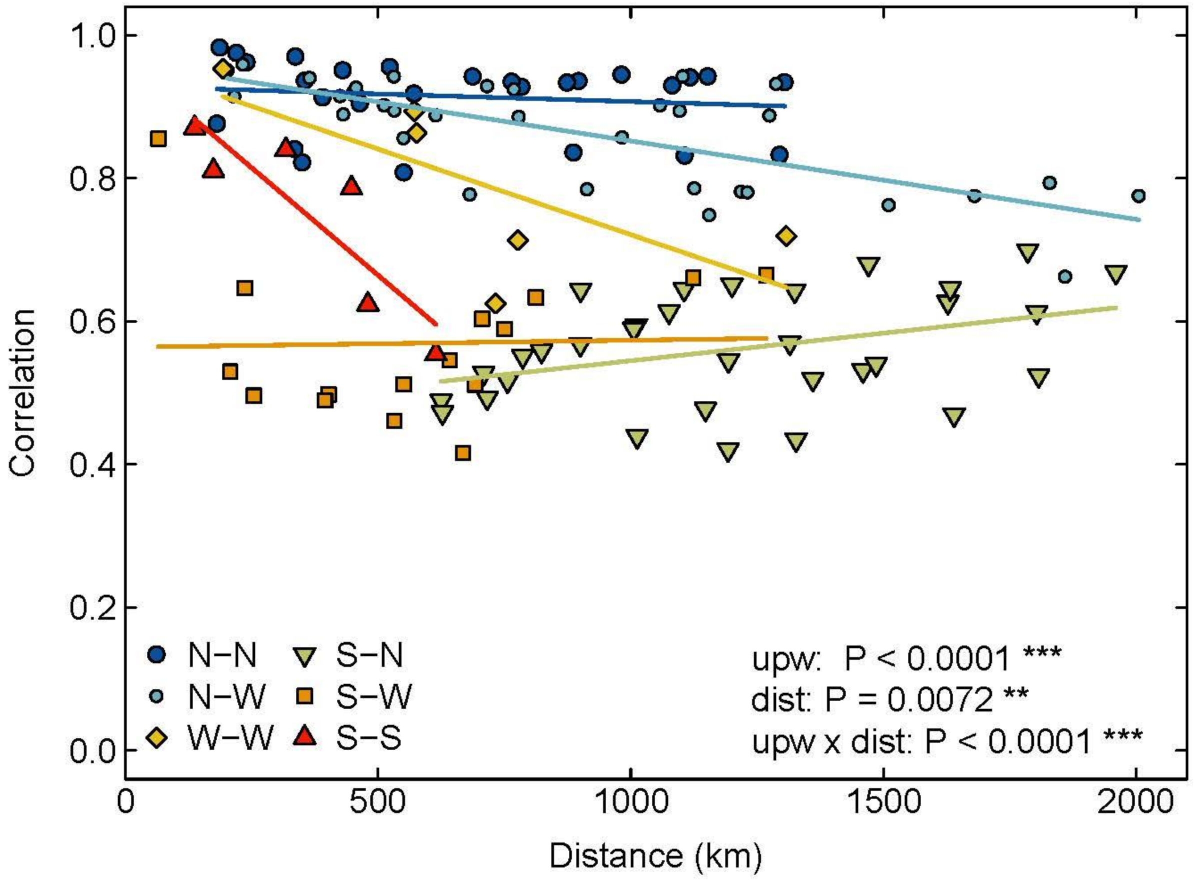

Figure S4.3. Pairwise wavelet coherence and phase difference in temperature between sites as a function of their geographical distance. Columns represent mean coherence (a-d) and phase difference spread measured in standard deviations (e-h). For reference, sites that experience phase synchrony will be characterized by a near zero standard deviation in phase difference. Rows represent all (2-889 days), annual (300-400 days), monthly (15-45 days) and weekly (2-10 day) periods. Data points are coded by color and shape based on each paired sites' upwelling condition combination. Strong upwelling pairs (S-S) are represented in red triangles, Strong-Weak upwelling pairs (S-W) are represented in orange squares, Weak upwelling pairs (W-W) are represented in yellow diamonds, No-Weak upwelling pairs (N-W) are represented in small light blue circles, No upwelling pairs (N-N) are represented in large dark blue circles, and Strong-No upwelling pairs (S-N) are represented in green inverted triangles. *P*-values are based on ANCOVA relating average pairwise coherence or standard deviation of phase difference in temperature between sites to their upwelling condition and geographical distance.

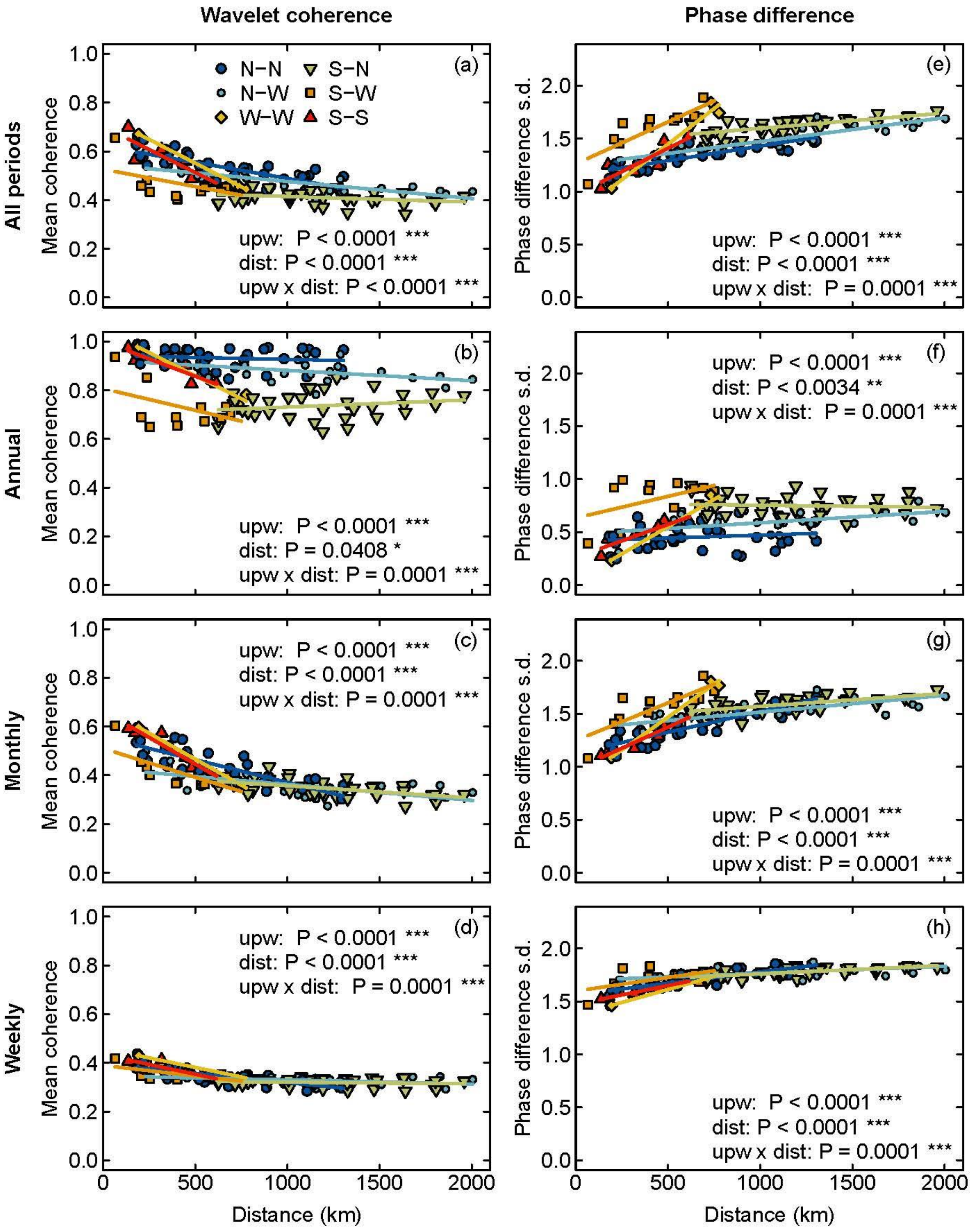

Wavelet coherence
Phase difference
All periods
Annual
Monthly
Weekly
Mean coherence
Phase difference s.d.
N−N
N−W
W−W
S−N
S−W
S−S
(a)
upw: P < 0.0001 ***
dist: P < 0.0001 ***
upw x dist: P < 0.0001 ***
(b)
upw: P < 0.0001 ***
dist: P = 0.0408 *
upw x dist: P = 0.0001 ***
(c)
upw: P < 0.0001 ***
dist: P < 0.0001 ***
upw x dist: P = 0.0001 ***
(d)
upw: P < 0.0001 ***
dist: P < 0.0001 ***
upw x dist: P = 0.0001 ***
(e)
upw: P < 0.0001 ***
dist: P < 0.0001 ***
upw x dist: P = 0.0001 ***
(f)
upw: P < 0.0001 ***
dist: P < 0.0034 **
upw x dist: P = 0.0001 ***
(g)
upw: P < 0.0001 ***
dist: P < 0.0001 ***
upw x dist: P = 0.0001 ***
(h)
upw: P < 0.0001 ***
dist: P < 0.0001 ***
upw x dist: P = 0.0001 ***
Distance (km)
Distance (km)
